# Aspiration in lethal drug abuse—a consequence of opioid intoxication

**DOI:** 10.1007/s00414-020-02412-y

**Published:** 2020-09-14

**Authors:** Johannes Nicolakis, Günter Gmeiner, Christian Reiter, Monika Heidemarie Seltenhammer

**Affiliations:** 1 Center for Forensic Medicine, Medical University of Vienna, Sensengasse 2, A-1090 Vienna, Austria; 2 Seibersdorf Laboratories, Campus Seibersdorf, A-2444 Seibersdorf, Austria

**Keywords:** Drug abuse, Opioid-related deaths, Drug-associated fatal aspiration, Emesis, Breath depression, Aspiration

## Abstract

**Aims:**

The primary objective of this study was to investigate whether the fatalities of opioid abuse are not only related to respiratory depression but also as a result of other side effects such as emesis, delayed gastric emptying, a reduction of the cough reflex, and impaired consciousness leading to the aspiration of gastric contents, a finding regularly observed in drug-related deaths.

**Design:**

A retrospective exploratory study analyzing heroin/morphine/methadone-related deaths submitted to court-ordered autopsy.

**Setting:**

Center for Forensic Medicine, Medical University of Vienna, Austria (2010–2015).

**Participants:**

Two hundred thirty-four autopsy cases were included in the study: morphine (*n* = 200), heroin (*n* = 11), and methadone (*n* = 23) intoxication.

**Findings:**

Analyses revealed that 41.88% of all deceased showed aspiration of gastric contents with equal gender distribution (*p* = 0.59). Aspiration was more frequent in younger deceased (*χ*^2^ = 8.7936; *p* = 0.012) and in deceased with higher body mass index (BMI) (*χ*^2^ = 6.2441; *p* = 0.044). Blood opioid concentration was lower in deceased with signs of aspiration than in non-aspirators (*p* = 0.013). Toxicological evaluation revealed a high degree of concomitant substance abuse (91%)—benzodiazepines (61.6%) and/or alcohol (21.8%).

**Conclusions:**

There are lower opioid concentrations in deceased with signs of aspiration, a fact which strongly points to aspiration as alternative cause of death in opioid-related fatalities. Furthermore, this study highlights the common abuse of slow-release oral morphine in Vienna and discusses alternative medications in substitution programs (buprenorphine/naloxone or tamper-resistant slow-release oral morphine preparations), as they might reduce intravenous abuse and opioid-related deaths.

## Introduction

Illicit substance abuse still continues to represent a widespread public health problem with the likelihood of a fatal outcome [1]. Opioid intoxication is the major cause of illegal-drug-associated deaths in Austria [2, 3]. While respiratory depression is the primary mechanism of death in opioid overdoses [4], previous studies frequently identified opioid concentrations generally considered to be non-lethal in a non-tolerant population, providing a strong indication of a different cause of death [5, 6]. Clinical expertise suggests that asphyxiation through aspiration is a considerable factor in drug-related deaths, as aspiration of gastric contents is a common complication of drug use [7–15]. Previous studies have found aspiration rates between 8.5 and 66% in deceased drug users [16, 17].

In terms of opioid-induced aspiration, this phenomenon is attributable due to 4 major mechanisms: (i) emesis, (ii) delayed gastric emptying, (iii) reduction of the cough reflex, and (iv) impaired consciousness.

Emesis is a common side effect in opioids [18]. The reported incidence rates of opioid-induced nausea and vomiting range from 8.3 to 40% in prescription opioids using therapeutic doses to control pain [19–22]. In a case series on acute intoxication in the hospital emergency department, Liakoni, Dolder, Rentsch, and Liechti [23] reported that 18% of all patients reported nausea or vomiting, while 27% of the patients had consumed opioids. Gender is reported to be a risk factor for opioid-induced emesis, with females being more prone to nausea or vomiting [24, 25]. The incidence of emesis decreases as age increases [26, 27]. Furthermore, opioids are known to reduce the cough reflex and have been used as potent antitussive agents for centuries (Mudge, 1778; Woolf and Rosenberg, 1962; Morice et al., 2007). Another important precondition for aspiration is the impaired consciousness induced by opioids.

In Austria, Heroin was the main cause of opioid-related deaths [2, 3, 28] until the use of slow-release oral morphine became commonplace as drug substitution medication, and became the most frequently abused opiate during the early 2000s [29–32]. Although originally intended to be orally consumed, substance abuse involving slow-release oral morphine involves it being injected after the morphine has been extracted by crushing and heating [33–37]. Moreover, polydrug abuse is a widespread problem given that most opioid addicts consume additional substances, especially other central nervous system depressants such as alcohol and/or benzodiazepines [38–40]—all of them increasing the risk of a potentially fatal side effect of opioids such a respiratory depression. The prevalence of benzodiazepine co-abuse ranges between 50 and 70% in heroin, methadone and buprenorphine users [41]. Studies by Aeschbach Jachmann, Jagsch, Winklbaur, Matzenauer, and Fischer [42] and Zeidler [32] found that benzodiazepines are often co-prescribed by general practitioners in Vienna in addition to substitution medication. This paper addresses the significance of aspiration of gastric contents in drug-related deaths. Furthermore, this study provides an overview of opioid-related deaths in Vienna between 2010 and 2015.

## Methods

### Study design

This exploratory, retrospective study evaluated the impact of aspiration on drug-related deaths, performed in accordance with the principles of the Declaration of Helsinki (Version Fortaleza 2013). The protocol was submitted to the Ethical Committee of the Medical University of Vienna (ECS 1003/2016).

### Data collection and definitions

All autopsy cases with blood samples that tested positive for drugs during a court-ordered autopsy at the Center for Forensic Medicine (formerly Department of Forensic Medicine) of Vienna Medical University from 2010 to 2015 were retrospectively reviewed. Suitable cases were identified by using the annual drug-related death reports submitted by the Center for Forensic Medicine to the Austrian Ministry of Health. These include all deaths where drugs were detected at some point, but not necessarily where the substances played a direct role in causing the death. As a result, autopsy, histology, toxicology, and police records were reviewed in detail to ascertain whether it was a drug-related death or whether death was due to other reasons. Drug-related deaths were determined by means of the circumstances of death, police investigations, and autopsy pathology findings.

Out of 2538 autopsy cases in the study period, 417 were considered to be drug related, while only 288 were opioid related. Fifty-four of these were excluded due to different reasons: non-drug-related cause of death (trauma, sepsis, *n* = 12), non-usable toxicology (long ICU admittance, decomposition, *n* = 18), premature birth (*n* = 1), or other drugs (*n* = 3) were causal for death. Additionally, all opioid-related deaths in which heroin, methadone, or morphine were not detected were excluded for comparability (*n* = 20). A total of 234 cases were included in this study and corresponding histological slides were re-examined for signs of aspiration.

#### Aspiration

Aspiration was categorized both (i) macroscopically (as fulminant (++), slight (+), or none (−)) and (ii) histologically (as particulate (++), gastric juice only (+), or none (−)). In terms of macroscopic staging, fulminant aspiration was defined as total occlusion or significant amounts of chyme in the trachea or main bronchi. No evidence of gastric contents in the respiratory system was classified as non-aspiration. All other cases were classified as slight aspiration. Regarding histological staging, particulate aspiration was defined as being the presence of skeletal muscle cells, cellulose, or material with double refraction in polarized light. Aspiration of only gastric juice was defined as a lack of particular matter and all of the following: signs of acidic digestion (edema, congestion, hemorrhage, degeneration of bronchiolar lining cells, and alveolar type I and II cells followed by alveoli filled with polymorphonuclear neutrophils and fibrin), local inflammatory reactions, intra-alveolar hemorrhage, and bacterial colonization.

#### Time of agony

Time of agony was determined in 90 cases (84 morphine, 6 heroin) using the method described by Vycudilik [43], using the quotient of the morphine concentration in the medulla oblongata and the brainstem (C_med_/C_cereb_). In cases with C_med_/C_cereb_ ratios < 0.8, death occurred within 1 h, intermediate C_med_/C_cereb_ ratios between 0.8 and 2.0 indicate survival for at least some hours, while C_med_/C_cereb_ values > 2.0 indicate survival for over 6 h.

### Toxicology

Toxicological analyses were conducted either at the Division of Medical-Chemical Laboratory Diagnostics at the Medical University of Vienna or at Seibersdorf Laboratories. Samples were analyzed with a general unknown screening method using either gas chromatography mass spectrometry, liquid chromatography tandem-mass spectrometry, or liquid chromatography high-resolution mass spectrometry. Findings were then confirmed and quantified by specific testing.

Deceased were attributed to heroin use when any matrix tested positive for morphine and 6-Monoacetylmorphine (6-MAM). Deceased who tested positive for morphine, but not for 6-MAM, were attributed to morphine administration.

Alcohol consumption was considered positive when the quantity exceeded 0.4‰ so as to avoid false positives due to possibility of the post-mortem formation of ethanol [44].

With respect to benzodiazepines, all of these individually detected drugs were grouped into the single drug class “benzodiazepines,” as it was not possible in some cases to ascertain whether the presence of two or more benzodiazepines detected at autopsy were due to the administration of two drugs or one drug and its metabolite (e.g., positive for diazepam and oxazepam). The occurrence of other opioids or illicit drugs was recorded in a separate data field for each substance found. Prescription drugs were also grouped into psychiatric and non-psychiatric drugs. Psychiatric drugs were subdivided into antidepressants, anticonvulsants, antipsychotics, and non-benzodiazepine sedatives.

### Group allocations

#### Fulminant aspiration/slight aspiration/non-aspiration

Deceased with fulminant macroscopic aspiration were assigned to the fulminant aspiration group. Deceased with either slight macroscopic aspiration or no macroscopic aspiration but positive histologic aspiration were assigned to the slight aspiration group. All other deceased were assigned to the non-aspiration group.

#### Monodrug/polydrug

As polydrug abuse is very common [40, 45], and interactions with opioid pharmacodynamics and kinetics may be present [41, 46], all deceased were divided into monodrug and polydrug groups. Deceased with a proven record of alcohol, benzodiazepine, other opioids, other illicit drugs, or psychiatric medication, in addition to the primary opioids, were assigned to the polydrug group.

#### Specific co-ingestion: alcohol/benzodiazepines

In order to further investigate the possible interactions of alcohol and/or benzodiazepines on aspiration or opioid levels, all deceased were additionally divided into four subgroups: opioid without alcohol or benzodiazepines, opioid with alcohol, opioid with benzodiazepines, and opioid with both alcohol and benzodiazepines.

### Statistical analysis

Data values in descriptive statistics were expressed as means, median, minimum, and maximum levels, as all collected continuous variables were not normally distributed. The Shapiro-Wilk test was used to determine the normality of continuous variables. Categorical variables were expressed as frequencies and percentages.

Given the exploratory nature of the data, no correction for multiple testing was made [47], except for post hoc tests. In order to describe group differences in aspiration depending on the extent and type of drug abuse, comparisons were made using the Wilcoxon rank sum test between two groups, and the Kruskal-Wallis test for three groups, with post hoc analysis using the Conover-Iman test. Categorical variables were analyzed using Fisher’s exact test for two groups, and the Fisher-Freeman-Halton test to compare three groups. Correlations were analyzed using the Spearman’s rank correlation coefficient. All tests were two-sided, and a *p* value of less than 0.05 was deemed to indicate statistical significance. All analyses were performed using R version 3.3.0 with RStudio version 0.99.902 statistics software.

## Results

### Demographic characteristics

Opioid-related fatalities in Vienna were predominantly males, making up 76% of all deceased), as shown in an overview in Table 1. We found no significant difference for sex of subjects in any tested main variable.

**Table 1:** Demographic characteristics of morphine/methadone/heroin-related deaths by aspiration group

Aspiration type	*n*	Age (years)	Sex	BMI (kg/m^2^)	Injection sites	CPR	Stomach contents
		Median (range)	M/F %	Median (range)	%	%	%
All	234	33 (16–80)	76/24	24.8 (13.5–54.5)	48.3	32.5	70.9
Fulminant A.	31	30 (19–55)	74/26	25.6 (17.9–42.3)	48.4	32.3	100
Slight A.	67	33 (20–54)	79/21	25.4 (17.4–41.4)	50.7	32.8	77.6
Non-Aspiration	136	35 (16–80)	76/24	23.8 (13.5–54.5)	47.1	32.4	61

**Fig. 1:**
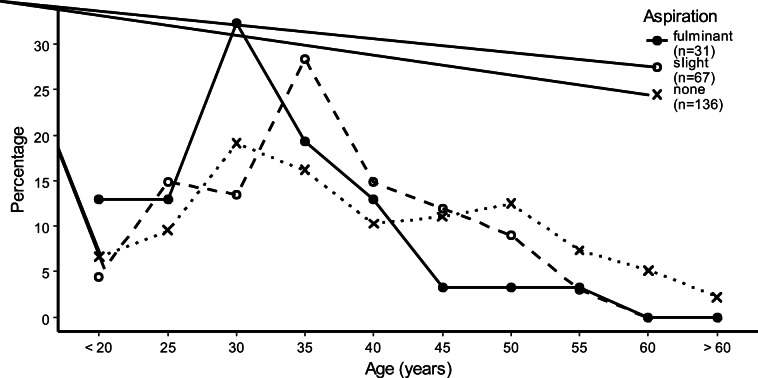
Frequency distributions of age in opioid-related deaths comparing fulminant, slight, and non-aspiration

The median age of the deceased was 33 years (range 16 to 80 years old). There was a significant age difference between the aspiration groups (Kruskal-Wallis test: *χ*^2^ = 8.7936, df = 2, *p* = 0.012). Post hoc comparisons using the Conover-Iman test indicated that fulminant aspirators were significantly younger than slight and non-aspirators, being on average 3 to 5 years younger (30 vs. 33 years, *t* = − 1.8886, *p* = 0.03, and 30 vs. 35 years, *t* = − 2.9696, *p* = 0.002). However, the age of non-aspirators did not significantly differ from the age of slight aspirators, as also shown in Fig. 1 and Table 1.

We found no difference in aspiration types between the sexes (Fisher-Freeman-Halton test: *p* = 0.818). Median body mass index (BMI) was 24.8 kg/m^2^ (range 13.5–54.5 kg/m^2^) and revealed a significant difference between aspiration groups (Kruskal-Wallis test: *χ*^2^ = 6.2441, df = 2, *p* = 0.044). Non-aspirators had a lower BMI than fulminant and slight aspirators (Conover-Iman test: BMI 23.8 vs. 25.6, *t* = 2.1771, *p* = 0.015, and BMI 23.8 vs. 25.4, *t* = 1.7725, *p* = 0.039). Additionally, there was a statistically significant difference between the sexes, with males having a higher BMI on average (25 vs. 23.5, Wilcoxon rank sum test: *W* = 4050, *p* = 0.047). A significant difference between stomach contents at autopsy and aspiration level was found (Fisher-Freeman-Halton test: *p* < 0.001). Fulminant aspirators had significantly more often filled stomachs than slight (Fisher’s exact test: *p* = 0.002) or non-aspirators (Fisher’s exact test: *p* < 0.001) and slight aspirators also had significantly more often filled stomachs than non-aspirators (Fisher’s exact test: *p* = 0.026).

### Resuscitation (CPR)

In order to ascertain whether resuscitation causes artificial aspiration, a *χ*^2^ test was performed, which revealed no significance differences in aspiration frequency (Table 1, *χ*^2^ = 0.0055, df = 2, *p* = 0.997), indicating that CPR has no effect on aspiration or aspiration level.

### Toxicology

#### Drug prevalence/polydrug use

In 91% of the deceased, more than one drug was present. There was no difference in aspiration frequency for polydrug use (Fisher-Freeman-Halton test: *p* = 1.0). Drug prevalence by aspiration group is shown in Table 2.

**Table 2:** Drug prevalence in morphine/methadone/heroin-related deaths by aspiration group

Variable	All (*n* = 234)	Fulminant (*n* = 31)	Slight (*n* = 67)	None (*n* = 136)
Morphine	200 (85.5)	24 (77.4)	62 (92.5)	114 (83.8)
Heroin	11 (4.7)	1 (3.2)	3 (4.5)	7 (5.1)
Methadone	23 (9.8)	6 (19.4)	2 (3)	15 (11)
Alcohol	51 (21.8)	5 (16.1)	15 (22.4)	31 (22.8)
Benzodiazepines	148 (63.2)	19 (61.3)	44 (65.7)	85 (62.5)
Other opioids	39 (16.7)	6 (19.4)	16 (23.9)	17 (12.5)
Tramadol	12 (5.1)	3 (9.7)	5 (7.5)	4 (2.9)
Dihydrocodein	8 (3.4)	2 (6.5)	3 (4.5)	3 (2.2)
Codein	17 (7.3)	1 (3.2)	6 (9)	10 (7.4)
Levorphanol	1 (0.4)	0 (0)	0 (0)	1 (0.7)
Oxycodon	1 (0.4)	0 (0)	1 (1.5)	0 (0)
Fentanyl	1 (0.4)	0 (0)	1 (1.5)	0 (0)
Other illicit drugs	58 (24.8)	7 (22.6)	15 (22.4)	36 (26.5)
Cocaine	43 (18.4)	4 (12.9)	11 (16.4)	28 (20.6)
Amphetamine	10 (4.3)	2 (6.5)	3 (4.5)	5 (3.7)
Methamphetamine	4 (1.7)	1 (3.2)	3 (4.5)	0 (0)
MDMA	7 (3)	1 (3.2)	0 (0)	6 (4.4)
THC	6 (2.6)	0 (0)	2 (3)	4 (2.9)

The results are presented as *n* (%)

##### Opioids

In the vast majority of cases, morphine (85.5%) was found to be the primary opioid. Methadone accounted for 9.8% of cases, while heroin could only be detected in 4.7% of all deceased. Methadone users show a significantly higher frequency of fulminant (26%) to slight aspiration (8%) than morphine or heroin users (Fisher-Freeman-Halton test: *p* = 0.019).

##### Alcohol

Alcohol was detected in 21.8% of all deceased. No statistically significant difference was found in consumption rates for aspiration group (Fisher-Freeman-Halton test: *p* = 0.768), primary opioid (Fisher-Freeman-Halton test: *p* = 0.416), or sex (Fisher’s exact test: *p* = 0.351). Among alcohol consumers, the median blood alcohol concentration was 1.22‰ (range 0.40 to 4.75‰). While ethanol concentration seems different between groups, no statistically significant difference in median blood alcohol concentration was found for sex (male: 1.19‰; female: 1.78‰; Wilcoxon rank sum test: *W* = 222.5, *p* = 0.415), aspiration type (fulminant: 1.22‰; slight: 1.48‰; non-aspiration: 1.17‰; Kruskal-Wallis test: *χ*^2^ = 0.5313, df = 2, *p* value = 0.767) nor primary opioid (heroin: 0.51‰; morphine: 1.37‰; methadone 1.69‰; Kruskal-Wallis test: *χ*^2^ = 4.6341, df = 2, *p* = 0.098), probably due to sample size effects.

##### Benzodiazepines

The majority of the deceased used benzodiazepines (61.3%). There was no difference between, aspiration groups (Fisher-Freeman-Halton test: *p* = 0.907), or primary opioid groups (Fisher-Freeman-Halton test: *p* = 0.405). Both alcohol and benzodiazepines were detected in 12.3% of all deceased. Benzodiazepine users were older than non-benzodiazepine users (median 35 years vs. 31 years, Wilcoxon rank sum test: *W* = 5360, *p* = 0.044).

##### Other illicit drugs

Cocaine (18.4%), amphetamine (4.3%), methamphetamine (1.7%), MDMA (3%), and THC (2.6%) were detected in addition to opioids. Heroin users seemed to take other illicit drugs more often (55%) than morphine (23.5%) or methadone users (22%), although this was not significant (Fisher-Freeman-Halton test: *p* = 0.079). No difference in consumption rates was found between aspiration groups (Fisher-Freeman-Halton test: *p* = 0.824), except for methamphetamine, which seemed to be associated with slight aspiration (Fisher-Freeman-Halton test: *p* = 0.035).

##### Intravenous abuse

The presence of recent injection sites as sign of intravenous drug abuse did not differ between aspiration groups (Fisher-Freeman-Halton test: *p* = 0.897), but morphine users (53.5%) were found to practice intravenous abuse more often than methadone users (17.4%) (Fisher-Freeman-Halton test: *p* < 0.001). Benzodiazepine use was also associated with recent injection sites (Fisher’s exact test: *p* = 0.002). Deceased with recent injection sites were statistically significant younger than those without (median 31 vs. 35 years, Kruskal-Wallis test: *χ*^2^ = 5.4017, df = 1, *p* = 0.020). Table 3 shows drug prevalence by recent injection sites. No statistically significant differences in the number of deceased with or without recent injection sites were found for sex, BMI, stomach fillings, CPR, alcohol usage, or polydrug abuse.

**Table 3:** Drug prevalence by recent injection sites

Injection	All (*n* = 234)	Yes (*n* = 113)	No (*n* = 121)
Morphine	183 (78.2)	100 (88.5)	83 (68.6)
Heroin	28 (12.0)	9 (8.0)	19 (15.7)
Methadone	23 (9.8)	4 (3.5)	19 (15.7)
Alcohol	51 (21.8)	22 (19.5)	29 (24.0)
Benzodiazepines	148 (63.2)	83 (73.5)	65 (53.7)
Other opiates	39 (16.7)	16 (14.2)	23 (19.0)
Tramadol	12 (5.1)	7 (6.2)	5 (4.1)
Dihydrocodein	8 (3.4)	5 (4.4)	3 (2.5)
Codein	17 (7.3)	3 (2.7)	14 (11.6)
Levorphanol	1 (0.4)	0 (0.0)	1 (0.8)
Oxycodon	1 (0.4)	1 (0.9)	0 (0.0)
Fentanyl	1 (0.4)	1 (0.9)	0 (0.0)
Other illicit drugs	58 (24.8)	26 (23.0)	32 (26.4)
Cocaine	43 (18.4)	23 (20.4)	20 (16.5)
Amphetamine	10 (4.3)	3 (2.7)	7 (5.8)
Methamphetamine	4 (1.7)	1 (0.9)	3 (2.5)
MDMA	7 (3.0)	3 (2.7)	4 (3.3)
THC	6 (2.6)	1 (0.9)	5 (4.1)

The results are presented as *n* (%)

#### Morphine concentrations in morphine users

##### Concentration of free morphine in blood

Table 4 shows the mean, median, range, and upper percentiles of concentrations of free morphine in the blood by aspiration type. There was a significant difference in morphine blood concentration between the three aspiration groups (Kruskal-Wallis test: *χ*^2^ = 7.9187, df = 2, *p* = 0.019); fulminant and slight aspirators had lower concentrations than non-aspirators (Conover-Iman test: *t* = − 2.2564, *p* = 0.013 and *t* = − 2.2569, *p* = 0.013).

**Table 4:** Free morphine blood concentration (μg/ml) by aspiration type

Aspiration type	*n*	Free morphine (μg/ml)			Upper percentiles (μg/ml)	
		Mean	Median	Range	75th	90th
All	200	0.94	0.45	0.01–14.00	0.90	1.87
Fulminant A.	24	0.47	0.26	0.05–1.90	0.79	1.04
Slight A.	62	0.64	0.37	0.01–4.30	0.73	1.24
Non-Aspiration	114	1.21	0.57	0.02–14.00	1.06	2.00

**Fig. 2:**
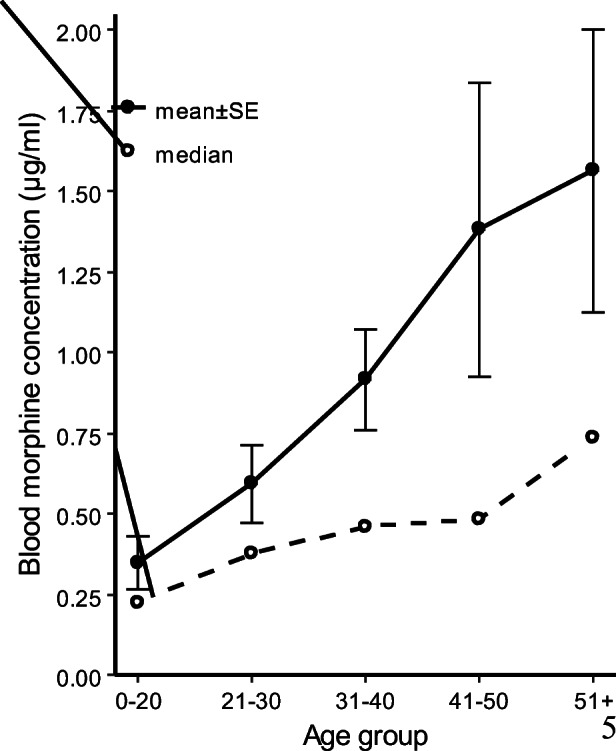
Variations in mean and median blood concentrations of free morphine by age. SE = Standard error

**Fig. 3:**
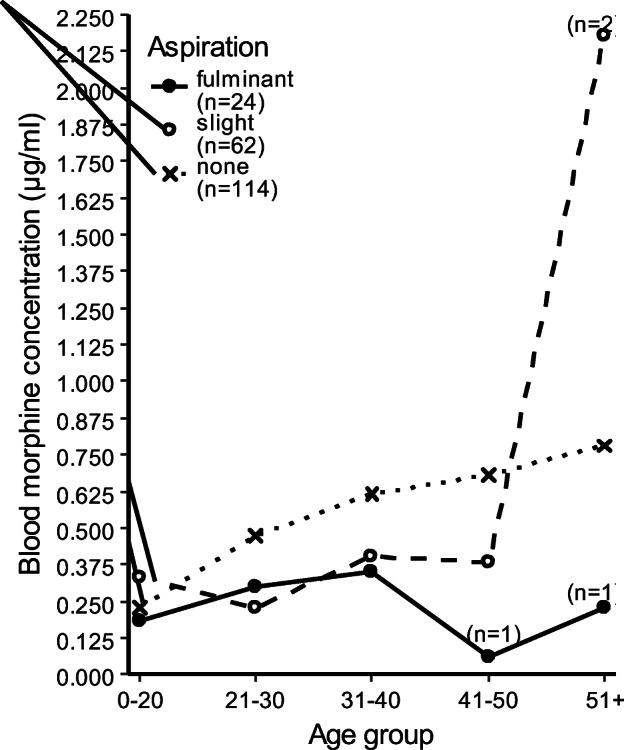
Variations in median blood concentrations of free morphine by age and aspiration type. Free morphine blood concentration in morphine-only deaths and polydrug users

Although only a negligible correlation with age was found (Spearman’s rank correlation coefficient: *ρ* = 0.213, *p* = 0.002), the line graph in Fig. 2 illustrates an association between age and free morphine blood concentration. A significant concentration difference was found between the age groups as used in Fig. 2 (Kruskal-Wallis test: *χ*^2^ = 13.382, df = 4, *p* = 0.01). Figure 3 shows median morphine blood concentration by age group and aspiration type. There was no difference in median morphine blood concentration between deceased with or without recent injection sites (Kruskal-Wallis test: *χ*^2^ = 0.3133, df = 1, *p* = 0.576).

Table 5 compares the concentrations of free morphine in the blood between mono-intoxications (morphine only) and polydrug deaths. Median concentrations in monodrug deaths were significantly lower than in polydrug deaths (0.19 vs. 0.48 μg/ml, Wilcoxon rank sum test: *W* = 1233, *p* = 0.021). Median morphine concentrations in morphine-only deaths were not significantly different in the aspiration groups (Kruskal-Wallis test: *χ*^2^ = 1.4195, df = 2, *p* = 0.492), but there were significant differences for polydrug deaths (Kruskal-Wallis test: *χ*^2^ = 6.9782, df = 2, *p* = 0.030). Fulminant and slight aspirators had lower median morphine concentrations than non-aspirators (Conover-Iman test: *t* = − 1.9005, *p* = 0.030 and *t* = − 2.3004, *p* = 0.011).

**Table 5:** Free morphine blood concentration (μg/ml) by aspiration type and mono- or polydrug death

Aspiration	Circumstances	N*n*	Free morphine (μg/ml)			Upper percentiles (μg/ml)	
			Mean	Median	Range	75th	90th
All	Morphine only	20	0.78	0.19	0.01–7.60	0.49	1.86
	Polydrug death	180	0.96	0.48	0.01–14.00	0.90	1.87
Fulminant	Morphine only	3	0.13	0.13	0.05–0.22		
	Polydrug death	21	0.51	0.28	0.06–1.90	0.87	1.10
Slight	Morphine only	5	0.32	0.19	0.01–0.89	0.30	0.65
	Polydrug death	57	0.67	0.37	0.01–4.30	0.75	1.28
None	Morphine only	12	1.14	0.19	0.06–7.60	0.97	2.15
	Polydrug death	102	1.21	0.61	0.02–14.00	1.06	2.00

**Fig. 4:**
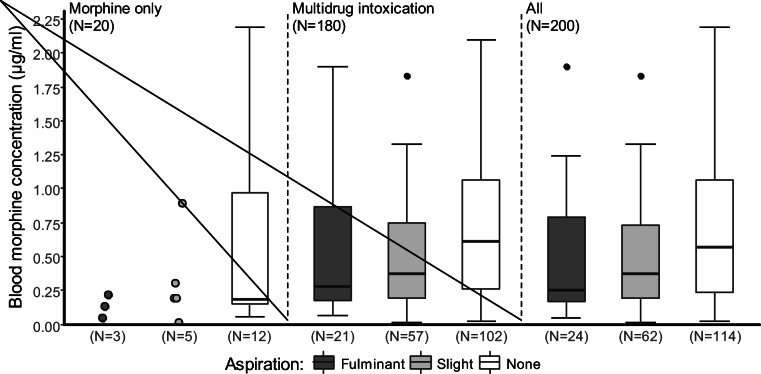
Box and whisker plots of free morphine concentrations in blood in morphine-related deaths comparing fulminant, slight and non-aspiration in morphine-only and polydrug deaths. Each box represents the inter-quartile ranges (IQR 25–75%). Medians are shown as horizontal lines (50%). Outliers above 2.25 μg/ml are not depicted (*n* = 12 in total)

A comparison of the blood concentration distributions of free morphine in morphine-only deaths and polydrug deaths by aspiration type is shown in Fig. 4.

##### Free morphine in blood and co-consumption of alcohol and/or benzodiazepines

**Fig. 5:**
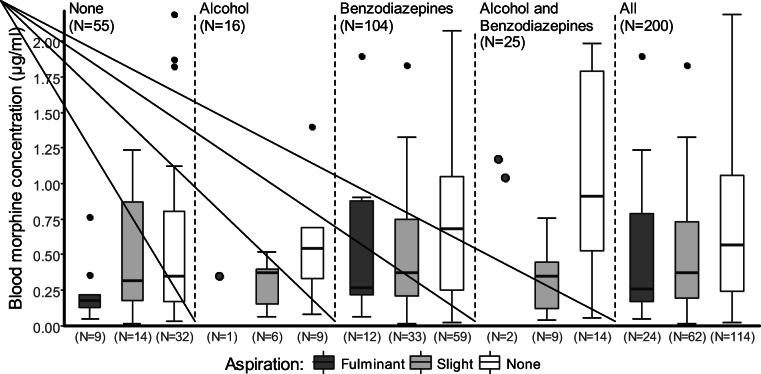
Box and whisker plots of free morphine concentrations in blood in morphine-related deaths comparing fulminant, slight, and non-aspiration and co-use of alcohol and/or benzodiazepines. Each box represents the inter-quartile ranges (IQR 25–75%). Medians are shown as horizontal lines (50%). Outliers above 2.25 μg/ml are not depicted (*n* = 12 in total)

The box and whisker plot in Fig. 5 depicts the concentration distributions of free morphine in the blood in morphine deaths without alcohol or benzodiazepines (Mo/N) (median 0.30 μg/ml), morphine and alcohol (Mo/A) (0.40 μg/ml), morphine and benzodiazepines (Mo/B) (0.53 μg/ml), or morphine, alcohol, and benzodiazepines (Mo/AB) (0.80 μg/ml). A significant difference between these four classifications was found (Kruskal-Wallis test: *χ*^2^ = 8.0246, df = 2, *p* = 0.045). Mo/N cases had a significantly lower concentration than Mo/B and Mo/AB cases (Conover-Iman test: *t* = − 1.9963, *p* = 0.024 and *t* = − 2.6867, *p* = 0.004). Mo/A and Mo/B cases did not differ from Mo/AB cases (Conover-Iman test: *t* = − 1.5682, *p* = 0.059 and *t* = − 1.4152, *p* = 0.079).

#### Blood concentrations in methadone and heroin users

Since the entire dataset contained only 23 methadone users and 11 heroin users, it was not possible to perform meaningful statistical tests on this group, especially when splitting data into the three aspiration groups (methadone: fulminant: *n* = 6, slight: *n* = 2, none: *n* = 15; heroin: fulminant: *n* = 1, slight: *n* = 3, none: *n* = 7, Table 6).

**Table 6:** Opioid concentration (μg/ml) by aspiration type in methadone and heroin users

Aspiration	Free methadone (μg/ml)				Free morphine (μg/ml)			
	*n*	Mean	Median	Range	*n*	Mean	Median	Range
All	23	0.86	0.42	0.06–5.60	11	0.36	0.28	0.06–1.12
Fulminant	6	0.58	0.43	0.13–1.60	1			0.08
Slight	2			0.41–1.00	3		0.16	0.14–1.12
None	15	1.00	0.41	0.06–5.60	7	0.36	0.29	0.06–0.83

### Time of agony

Brainstem and cerebellum morphine levels were available in 90 deceased (fulminant aspiration *n* = 17, slight aspiration *n* = 24, non-aspiration *n* = 49).

**Fig. 6:**
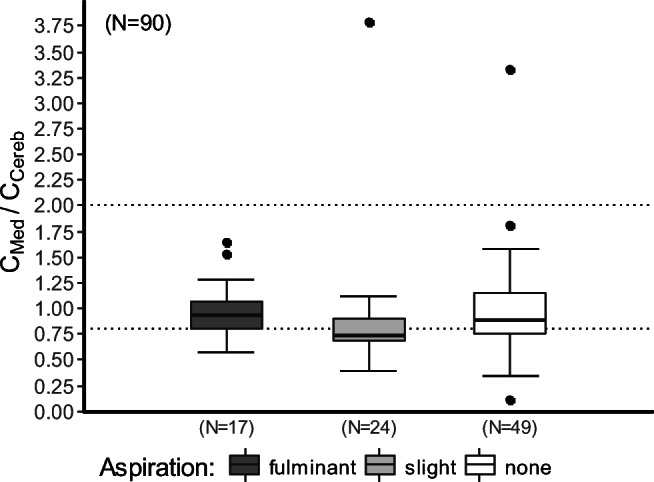
Box and whisker plots of C_Med_/C_Cereb_ in opiate-related deaths comparing fulminant, light and non-aspiration. The dotted lines illustrate the thresholds for death under an hour (C_Med_/C_Cereb_ < 0.8), death within 1 to 6 h (C_Med_/C_Cereb_ ≥ 0.8 and < 2.0) and death after at least 6 h (C_Med_/C_Cereb_ ≥ 2.0) [43]. Each box represents the inter-quartile ranges (IQR 25–75%); medians are shown as horizontal lines (50%)

A statistically significant difference between aspiration groups was found (Kruskal-Wallis chi-squared = 6.3542, df = 2, *p* = 0.042). Slight aspirators had a significantly lower C_med_/C_cereb_ ratio than fulminant (0.74 vs. 0.95, Conover-Iman test: *t* = 2.281599, *p* = 0.013) and non-aspirators (0.74 vs. 0.90, Conover-Iman test: *t* = − 2.238316, *p* = 0.014), whereas there was no difference between fulminant and non-aspirators (0.95 vs. 0.90, Conover-Iman test: *t* = 0.588312, *p* = 0.279), indicating a shorter time of agony in slight aspirators, also shown in Fig. 6 and Table 7. No statistically significant difference in C_med_/C_cereb_ ratio was found for injection sites (Kruskal-Wallis test: *χ*^2^ = 2.0331, df = 1, *p* = 0.154), multidrug use (Kruskal-Wallis test: *χ*^2^ = 1.7183, df = 1, *p* = 0.190) or age group (Kruskal-Wallis test: *χ*^2^ = 7.8054, df = 8, *p* = 0.453).

**Table 7:** C_Med_/C_Cereb_ ratio by aspiration

Aspiration	Cases	Mean	Median	Range
All	90	0.95	0.88	0.12–3.80
Fulminant	17	0.98	0.93	0.57–1.65
Slight	24	0.89	0.74	0.39–3.80
None	49	0.97	0.89	0.12–3.33

## Discussion

The present study was conducted as an exploratory study to investigate opioid-related deaths in Austria, particularly in connection with asphyxiation through aspiration. As such, statistics were compiled without correcting for inflation of alpha errors and should be treated as exploratory statistics only. Further confirmatory studies should be conducted in order to be able to draw a reliable conclusion about aspiration in opioid-related deaths. Since this was a retrospective study analyzing all suitable cases in the timeframe of 2010 to 2015, we were not able to use balanced group sizes regarding various factors (e.g., age, sex). Nevertheless, to control for some these factors, we divided our data into various subgroups (e.g., sex, polydrug consumption) and analyzed the data separately.

The aim of this study was to explore the significance of aspiration in opioid-related deaths. In this study, 41.88% of 234 deceased opioid users were found to have aspirated gastric contents, with 13.25% showing fulminant aspiration. This rate of aspiration lies between previous findings, which showed between 8.5 [16] and 66% [17]. In fulminant aspiration cases, cause of death was mostly stated as asphyxiation by aspiration, often in combination with respiratory depression, while slight aspiration and non-aspiration were classified as death by opioid-induced respiratory depression.

In contrast to the higher incidence of opioid-induced emesis in females reported in the literature [24–26], no difference was found in aspiration frequency between the sexes.

Interestingly, fulminant aspirators were younger than slight and non-aspirators. From this point of view, one might suggest that experience and tolerance might be an important factor in aspiration as the cause of death. If older drug-abusers are believed to have more experience, they might have developed a tolerance to opioid-induced emesis, while novice drug users might experience a higher stimulation of the chemoreceptor trigger zone in the area postrema and therefore be susceptible to a higher risk of morphine-induced emesis. This relation between age and aspiration is consistent with the finding that older deceased showed a higher morphine blood concentration.

Taken together, these results are consistent with a study conducted by Cepeda, Farrar, Baumgarten, Boston, Carr, and Strom [26], which showed that the risk of nausea and vomiting was lower in older opioid users. This could explain why, in the recent study, more instances of aspiration were found in younger people. In addition to this, older opioid users also have a higher risk of respiratory depression [26, 27], and thus might be more likely to die by respiratory depression caused by the opioid, rather than expiring as a result of aspiration.

Aspiration was more frequent in deceased with a higher BMI, which is consistent with published literature concerning perioperative aspiration risk [48, 49].

The occurrence of injection sites did not differ between aspiration groups, even though the intravenous administration of opioids is shown to induce a higher risk of nausea than is the case for oral administration [27]. This might be explained by the increased risk of respiratory depression in intravenous opioid use [27]. Additionally, there was an age difference in intravenous drug abuse, indicating that young people were more likely to practice intravenous drug administration than older people. Thus, the higher risk of emesis after intravenous opioid administration might be a possible explanation for higher incidences of aspiration in young deceased. Furthermore, the association between the use of benzodiazepines and intravenous opioid abuse could be an indicator for drug tolerance when oral administration or single drug use might show no sufficient opioid effect.

One of the main hypotheses in this study was that deceased who show evidence of aspiration, especially fulminant aspiration, have lower opioid blood concentrations. High opioid doses are expected to cause death directly by respiratory depression [4]. However, since there were also cases in which opioid concentrations generally considered to be non-lethal for a non-tolerant population were found, the cause of death might be attributable to factors other than respiratory depression.

As expected, results showed a lower morphine concentration in deceased with signs of aspiration than in deceased without aspiration of gastric contents. This effect was particularly evident when morphine was combined with other drugs, but not in morphine-only deaths. This suggests that the use of morphine with or without co-substances can result in different outcomes, not least because major interactions with alcohol and benzodiazepines are present [41, 50], and both can cause or increase emesis and respiratory depression [51].

Furthermore, the result that monodrug users and deceased without benzodiazepine use had a lower morphine blood concentration also suggests that concomitant substance abuse might increase with longer periods of morphine abuse. As the effect of morphine fades over time due to an increase in tolerance, users might increase the dosage as well as starting to take other drugs to increase the effect.

An alternative explanation of the results showing lower concentrations in the aspiration groups could be due to a longer time of agony during which the initial administered opioid concentration has decreased. To assess whether aspiration had an influence on the time of agony we compared the ratio of morphine concentration in the medulla oblongata and brainstem (C_Med_/C_Cereb_) as an indicator for time of agony [43]. The results showed no difference in time of agony between fulminant and non-aspirators while slight aspirators had a lower time of agony than both other groups. This indicates that the lower blood concentrations in fulminant aspirators compared with non-aspirators is not an effect of a longer time of agony, confirming the main hypothesis. Surprisingly, slight aspirators seem to have died significantly faster than both fulminant and non-aspirators, suggesting a high probability of agonal aspiration in these cases. Further studies should be conducted to investigate this question.

Although the aim of this study was to look at morphine-, methadone-, and heroin-related deaths, the study mainly focused on deceased with morphine intoxications, since the number of methadone and heroin users were too low to be able to draw meaningful conclusions. However, the ratio of fulminant to slight to non-aspiration was different in methadone users, with a higher ratio of fulminant aspirations than in morphine and heroin users. This is consistent with the study of Eder, Jagsch, Kraigher, Primorac, Ebner, and Fischer [52], which showed a higher incidence of vomiting in methadone maintenance treatment compared with slow-release oral morphine.

The low number of heroin users in this study might be attributable to the fact that heroin is metabolized to morphine. Thus, it may be the case that some of the deceased included in the morphine group were actually heroin users, but 6-MAM could not be detected anymore during autopsy. While techniques are described to distinguish heroin deaths from codeine deaths by using morphine-to-codeine ratios [53], these techniques are not useful when distinguishing heroin intake from slow-release oral morphine. An examination of vitreous humor could increase the accuracy of detecting heroin deaths under such circumstances [54, 55].

In general, the low number of deceased with methadone- and heroin-related deaths during the period of the study from 2010 to 2015 highlights the widespread use of slow-release oral morphine in Austria. With the increased use of morphine in substitution programs [29, 32], the illicit use of prescription opioids as alternative to heroin has increased over the past decade, including intravenous abuse [30].

## Conclusion

When taken together, the data suggests that aspiration occurred mainly in younger, overweight deceased with lower concentrations of morphine in the blood. By the same token, morphine blood concentration levels rise with age and the consumption of other drugs, especially benzodiazepines. Since the present study was conducted as an exploratory study, further studies should build on this information to focus on particular questions in more detail.
